# Promoting access to dental care in South London: adult patients’ perspectives

**DOI:** 10.1007/s10389-017-0821-4

**Published:** 2017-08-25

**Authors:** Sylviana Haji Moris, Orla Carty, Kristina L. Wanyonyi, Jennifer E. Gallagher

**Affiliations:** 1 Ministry of Health, Brunei Darussalam, Bandar Seri Begawan, Brunei; 2 Department of Orthodontics, University of Liverpool, Liverpool, UK; 3 King’s College London Dental Institute, Division of Patient and Population Health, Denmark Hill Campus, Bessemer Road, London, SE5 9RS UK; 4 University of Portsmouth Dental Academy, University of Portsmouth, Portsmouth, UK

**Keywords:** Access, Dentistry, Improving uptake, Barriers to dental care, Dental health, General dental practice

## Abstract

**Objective:**

To evaluate patients’ views on health service initiatives established to improve uptake of NHS primary dental care amongst adult patients in a socially deprived area, comparing practices with extended and regular contract capacity.

**Study design:**

Service evaluation and cross-sectional survey.

**Method:**

Questionnaire survey of patients attending a random sample of dental practices in three inner-metropolitan boroughs of south London following initiatives to improve access to dental care (across dental practices delivering regular and extended contracts for services) exploring attendance patterns and the influence and awareness of local initiatives to promote access.

**Results:**

Four hundred fifty adults across 12 dental practices completed questionnaires: 79% reported attending for routine and 21% for urgent care. Patients were most aware of banners outside practices, followed by dental advertisements in newspapers. Vouchers for free treatments were considered of the highest possible influence, followed by vouchers for reduced treatment costs and an emergency out-of-hours helpline. Awareness and influence were not aligned, and there was no evidence of difference by practice contract type whilst there were differences by age and type of attendance.

**Conclusion:**

The findings suggest that financial incentives and emergency services are considered the most influential initiatives for adult patients whose attendance patterns appear to be related to personal circumstances rather than merely being influenced by the provision of information.

## Introduction

Access to basic health services is a fundamental human right (World Health Organization 2013) and further emphasised as playing a role in addressing global inequalities (Marmot 2008). Access to health care is a complex and multi-dimensional concept and as a result there are many definitions. Guay (2004) considered access to be a supply and demand issue, encompassing both the availability of dental care as well as the willingness of the patient to seek care. More recently Harris (2013) proposed the construct of dental access as involving the following four concepts: opportunity for access, realised access (utilisation), equity and outcomes. Gulliford and Morgan (2003) describe the factors influencing access as multifaceted and the importance of research on barriers to access. Barriers to dental care have been well researched nationally (Finch et al. 1988; Hill et al. 2013; Kelly et al. 2000) and locally (Borreani et al. 2008, 2010), with Borreani et al. (2008) classifying barriers in older adults as ‘active’ or ‘passive’: active barriers include availability, accessibility, cost, fear and features of the dentist, whereas lack of perceived need is a passive barrier. Despite the research into barriers, there is a relative paucity of research on initiatives to improve the uptake of dental care (Gilbert et al. 2009), and the published literature tends to relate to small local initiatives or policy initiatives (Anderson and Morgan 1992).

In England, the uptake of dental care decreased between 2006 and 2008 following the introduction of a new dental contract in April of 2006. Steele (2009), in a review commissioned by the Secretary of State for Health, reported in 2009 that up to 24% of the population had difficulty in locating an NHS dentist, and 20% perceived poor quality of care from NHS dentists. A national ‘Dentistry Watch’ survey conducted in 2007 on 5212 patients found that 20% avoided treatment because of cost, while 50% of all NHS patients found NHS dental charges confusing (Commission for Patient and Public Involvement in Health 2007). It was evident that access to NHS dentistry needed to be improved, and this became a political imperative supported by resources (Department of Health 2009, 2011; Steele 2009). Locally, NHS Primary Care Trusts [PCTs] used the additional resources provided to increase the uptake of NHS dentistry to implement a series of initiatives to address dental uptake in metropolitan inner city area during 2009/10. Individual practices were encouraged to form targets and plans to improve the uptake by dental patients and increase the provision of dental care, which has then been compared with national data. A range of initiatives was funded by local PCTs to improve access including banners, advertisements in local newspapers, advertisements on buses and bus shelters, free toothpaste/-brush packs for new patients and cinema advertisements, as listed in Tables 2 and 5. Also some individual practices had further initiatives funded, including free check-up vouchers for new patients and a texting service to remind patients of appointments. Information-based initiatives to improve the uptake of dental care have been evaluated and shown to provide insight into both practitioner and patient perceptions, but with little impact on service uptake (Anderson and Morgan 1992). In a similar way the current study examined the impact of dental access initiatives on patients’ awareness and perceived influence in patients in inner city metropolitan areas of London. These boroughs have socially deprived and ethnically diverse populations, with poor dental uptake patterns (Gallagher et al. 2010). At the time, health service data suggested that 56% of the adults in Lambeth, 51% in Southwark and 59% in Lewisham had utilised an NHS dentist in the preceding 24 months based on the local resident population (NHS Digital 2009).

## Aim

To explore the views of adult patients attending primary dental care in inner city practice on initiatives to raise awareness and improve dental access.

## Methods

Dental practices with, and without, extended NHS capacity (referred to as extended and regular practices in this article) were identified and practice principals invited to participate in this research. NHS contracts were across the three boroughs of Lambeth, Southwark and Lewisham in South London. The study was designed to have a power of 80%, at the 5% significance level, to detect standardised differences of 0.25 and above between extended and regular contracts, for which a total of 256 respondents in each category were required. Therefore, 60 patients were requested per practice to account for a possible 25% non-response rate.

A questionnaire was developed for adult patients (16 years and over) attending participating dental practices. The self-completed questionnaire explored key areas: reasons for dental attendance; awareness of the range of local service initiatives to promote primary dental care during the past year; whether these initiatives were perceived to influence their behaviour to access NHS dental care; barriers to dental care; patient demography. The patient questionnaire was based on previous research: a study on marketing dentistry in Dudley by Anderson and Morgan (1992); the national adult dental health survey questionnaire (Kelly et al. 2000; NHS Digital 2011b); the Commission for Patient and Public Involvement in Health (2007).

The survey instrument consisted of 15 questions in three sections:

- Part I: Dental Attendance Pattern
- Part II: Awareness of Initiatives, Influence of Initiatives and Barriers to Care
- Part III: Information about the respondent

A five-point Likert scale ranging from ‘definitely yes’ to ‘definitely no’ was used as responses to the questions around ‘awareness’ of initiatives and the reverse of the scale was used on the questions around ‘influence’ of initiatives.

The patient questionnaire was tested through face-to-face piloting in two dental practices in Lambeth and Lewisham. The respondents were invited to report whether the questionnaire was understandable, and acceptable, as advocated by Bowling (2002). The questionnaire was found to be generally easy to complete and acceptable by the patients. However, based on patient feedback, it was necessary to reword the question relating to the *influence* of initiatives as these initiatives came, went and varied across the boroughs. It was clear that patients were interpreting the question on whether these *had* influenced them to include *would have* used them. Thus the question was amended to explore whether they had or would have changed their behaviour towards access to NHS dental care. This was undertaken with the view that the findings would inform future initiatives.

The questionnaire was administered by the practice staff to adult patients attending the participating practice in late spring 2010. There was a sign posted at the entrance and in the waiting area identifying that a survey was taking place. Adult patients were given an information sheet about the evaluation and asked if they would like to participate. Those who accepted were asked to complete the questionnaire survey in the waiting room and place it in a designated, sealed box at the reception for the research team; anonymity was assured. The researcher (SHM) made regular visits throughout the week to the participating dental practices to check the progress of the survey and collected completed questionnaires at the end of the week. Paper and computer records were coded by dental practice. The researcher was blind to the nature of the practice contract and unblinding did not occur until analysis was completed.

Quantitative analysis was undertaken using SPSS v21 following data input onto a computer. Descriptive, univariate and factor analyses were conducted to ascertain the awareness of initiatives and their influences on the patients based on socio-demography. Correlation between awareness and influence was measured using Pearson’s correlation coefficient. Significance was taken as ≥0.05.

## Results

### Participating practices

Practices were categorised by type of contract (category A, which had regular contracts, while category B had extended contracts) and borough (Lambeth, Southwark and Lewisham). Random sampling continued in each borough until 12 practices had been identified that were willing to participate. Twenty-five out of 37 dental practices invited refused to participate giving the following reasons: the principal dentist not available to speak or always in attendance, too busy, lack of interest and already participating in another study. Of the practices willing to participate in the research, seven had extended and five regular contracts.

### Patient respondents

The target size for the patient questionnaire was 512. As displayed in Table 1, a response of 88% (*n* = 450) was achieved. The majority had attended for a routine check-up (79%; *n* = 352), were female (61%, *n* = 253, white (63%, *n* = 282), from the regular contract practices (63%, *n* = 283) and fee paying (62%; 229). The largest age group was in the 35–44 year age band (21%, *n* = 89), with those aged over 75 years the smallest (2.4% *n* = 10).

**Table 1:** Practice and patient characteristics of respondents, *n* = 450

		Number	Per centage %
Practice characteristics			
Type of contract *n* = 450	Regular contract (category A)	283	62.9
	Extended contract (category B)	167	37.1
PCT *n* = 450	Lambeth	185	41.1
	Southwark	112	24.9
	Lewisham	153	34
Patient characteristics			
Sex *n* = 415	Male	162	39
	Female	253	61
Age group *n* = 415	16–24	48	11.6
	25–34	81	19.5
	35–44	89	21.4
	45–54	81	19.5
	55–64	61	14.7
	65–74	45	10.8
	75 and over	10	2.4
Ethnicity *n* = 413	White	282	63
	Asian	28	6
	Chinese	5	1
	Black	73	16
	Mixed ethnicity	25	6
	Other	4	1
NHS payment status *n* = 369	Yes	229	62.1
	No	138	37.4
	Other	2	0.5
Mobility of residence *n* = 362	More than once	42	11.6
	Once	76	21
	Never	237	65.5
	Other	7	1.9
Period with dental practice *n* = 447	More than 2 years	284	63.5
	1 to 2 years	65	14.5
	Less than a year	69	15.4
	First visit today	29	6.5
Current dental visit *n* = 445	Routine check-up/planned work/other	352	79.1
	Urgent	93	20.9
Main factor in choice of dentist *n* = 445	Location	200	44.9
	Reputation	125	28.1
	Both location and reputation	22	4.9
	Others	98	22
Past dental attendance *n* = 444	Less often or only when having trouble	124	27.9
	About the same	203	45.7
	More often	117	26.4

Only 7% (*n* = 29) of respondents reported being new patients to the dental practices. In contrast, almost two thirds (64%, *n* = 284) indicated that they had been with their dental practice for more than 2 years. Almost four out of five (79%; *n* = 352) adults reported attending a routine or planned appointment, with 21% (*n* = 93) attending for a dental emergency. Out of the 445 adults who responded to the question exploring the rationale for their choice of practice, location (45%, *n* = 200) was reported as the most influential factor, followed by reputation (28%, *n* = 125). Twenty-two per cent (*n* = 98) suggested other reasons for their choice of practice, the majority of whom reported that they always used this dental practice; this represented 19% of all respondents (*n* = 85). Only 5% (*n* = 22) reported both location and reputation as influential in their decision. In terms of location, being near home was the most common reason reported by 39% (*n* = 174).

The order in which factors influenced choice of dental practice was similar in both extended and regular contract practices, with a slightly higher proportion of respondents from practices with extended contracts (46%, *n* = 77) reporting location as important compared with regular contracts (44%, *n* = 123). Reputation was reportedly favoured by 31% (*n* = 85) of respondents from practices with established contract practices compared with 24% (*n* = 40) from those with extended contracts. Overall, although amongst both men and women, most people favoured location as a factor influencing the choice of practice significantly (*p* = 0.01); more men (57%, *n* = 92) selected location compared with women (37%, *n* = 92), whilst reputation was favoured by women (31%, *n* = 78 cf. 25%; *n* = 40).

### General patient awareness and influence of initiatives

Factor analysis was carried out to identify the constructs in the questionnaire confirming two factors; first their ‘awareness of Initiatives’ and second, ‘influence by initiatives’. The factor loading for each scale ranged between 0.9 and 0.7 for influence of initiatives and 0.7 and 0.57 suggest a good understanding of the questions by the participants. This is displayed in Table 2.

**Table 2:** Awareness and influence of initiatives to improve uptake of dental care

	Cronbach alpha	Factor loading	Mean	SD	N = (331)
Scale 1: awareness of initiatives (are you aware of or have you seen any of the following initiatives?)	0.879				
Banners outside dental practices (most aware)		0.566	2.89	1.256	331
Dental advertisements in newspapers		0.697	2.2	1.127	331
Dental article in a magazine		0.656	2.06	1.136	331
Dental leaflets/flyers		0.705	2.03	1.081	331
Dental advertisements on buses		0.624	1.92	1.064	331
Emergency out of hours helpline		0.665	1.91	1.086	331
Dental posters at bus stops		0.65	1.68	0.915	331
PCT pals		0.645	1.52	0.97	331
Free toothpaste packs for new patients initiatives?		0.622	1.52	0.929	331
Vouchers for reduced cost of treatment		0.696	1.36	0.832	331
Removable helpline card in a magazine		0.703	1.34	0.704	331
Vouchers for free treatment (least aware)		0.667	1.28	0.719	331
Scale 2: influence of initiatives (which of the following initiatives have or would have changed your behaviour towards access to NHS dental care?)	0.956				*N* = 334
Vouchers for free treatment (most influential)		0.743	2.31	1.338	334
Vouchers for reduced cost of treatment		0.75	2.42	1.326	334
Emergency out of hours helpline		0.799	2.71	1.236	334
Free toothpaste packs for new patients		0.784	2.91	1.304	334
Banners outside dental practices		0.761	2.95	1.239	334
Dental leaflets/flyers		0.891	3.07	1.166	334
PCT pals		0.828	3.1	1.217	334
Dental advertisements in newspapers		0.88	3.13	1.202	334
Dental article in a magazine		0.876	3.13	1.201	334
Removable helpline card in a magazine		0.863	3.14	1.222	334
Dental advertisements on buses		0.818	3.26	1.187	334
Dental posters at bus stops (least influential)		0.842	3.28	1.166	334

1. Scales are reversed with higher awareness marked by a higher mean score, whilst higher influence marked by a low mean score

2. Extraction method: principal component analysis

3. Rotation method: Oblimin with Kaiser normalisation

4. Reliability test: Cronbach alpha

Additionally, Table 2 highlights that respondents reported the highest *awareness* of banners outside dental practices followed by dental advertisements in newspapers. The *most influential* initiative was rated as vouchers for free treatments followed by vouchers for reduced treatment costs and banners outside dental practices, with the *least influential* being dental posters at bus stops. Awareness and influence were not aligned. For example, although patients were most aware of banners, they were only the 4th most influential out of the 12 initiatives examined. There were, however, differences in the overall awareness and influence of the initiatives between groups as shown in Table 3 (awareness) and Table 4 (influence).

**Table 3:** Univariate analysis of differences between patient and practice characteristics: awareness, *n* = 450

Awareness scale		Mean	SD	F(df)	*P* value
PCT *n* = 450	Lambeth	1.9	0.7	2.6 (330)	0.07
	Southwark	1.89	0.6		
	Lewisham	1.7	0.6		
Contract type *n* = 450	Regular	1.8	0.6	2.5 (330)	0.116
	Extended (additional capacity)	1.9	0.7		
Age group *n* = 415	16–24	1.9	0.7	0.7 (314)	0.621
	25–34	1.9	0.6		
	35–44	1.9	0.7		
	45–54	1.8	0.6		
	55–64	1.7	0.6		
	65–74	1.6	0.5		
	75 and over	1.6	0.5		
Ethnicity *n* = 413	Asian	1.7	0.6	0.9 (313)	0.453
	Black	1.9	0.7		
	White	1.8	0.6		
	Mixed	2.1	1.2		
	Chinese	1.7	0.8		
	Other	2.1	0.5		
Sex *n* = 415	Male	1.7	0.7	2.7 (311)	0.133
	Female	1.8	0.6		
Type of current care *N* = 445	Routine check-up/planned work/other	1.8	0.7		
	Urgent	1.7	0.5	4.9 (326)	**0.026**
Past dental attendance pattern *n* = 444	Urgent	1.6	0.6	4.8 (128)	**0.03**
	Regular	1.8	0.7		
Pay for NHS treatment *N* = 369	Yes	1.8	0.6	0.01 (275)	0.938
	No	1.8	0.7		

*Bold *p* values are statistically significant

The higher the score the more aware the group is of overall initiatives

**Table 4:** Univariate analysis of initiatives scale by patient and practice characteristics: influence, *n* = 450

Influence scale		Mean	S D	F(df)	*P* value
PCT *n* = 450	Lambeth	2.8	1.0	3.5 (331)	**0.032**
	Southwark	3.2	1.1		
	Lewisham	3.0	1.0		
Contract type *n* = 450	Regular	2.9	1.0	0.62 (333)	0.409
	Extended	3.0	1.0		
Age group *n* = 415	16–24	2.8	0.8	3.2 (6312)	**0.005**
	25–34	2.9	0.9		
	35–44	2.8	1.0		
	45–54	3.0	1.0		
	55–64	3.4	1.2		
	65–74	3.2	1.0		
	75 and over	3.8	1.0		
Ethnicity *n* = 413	Asian	2.7	1.1	2.4 (318)	**0.035**
	Black	2.6	1.1		
	White	3.2	1.0		
	Mixed	3.1	1.0		
	Chinese	3.2	0.3		
	Other	2.6	0.7		
Sex *n* = 415	Male	3.2	1.0	2.9 (316)	**0.004**
	Female	2.8	1.0		
Type of current care *N* = 445	Routine check-up/planned work	2.9	1.0	0.39 (331)	0.534
	Urgent	3.0	1.0		
Past dental attendance pattern *n* = 444	Urgent	2.9	1.0	0.39 (331)	0.144
	Regular	3.2	1.0		
Pay for NHS treatment *N* = 369	Yes	3.0	1.0	1.6 (281)	0.206
	No	2.9	1.0		

*Bold *p* values are statistically significant

The lower the score the more influenced the group is of overall initiatives

The findings presented in Tables 3 and 4 suggest that there were no significant differences in awareness and influences of initiatives between the two contract types. There were, however, significant differences by patient group: respondents attending for routine appointments or having a history of regular attendance reported significantly higher overall *awareness* of initiatives compared with those attending for emergency care (*p* = 0.03). In relation to the *influence* of initiatives, females were significantly more influenced than males (*p* = 0.004), Lambeth than the other boroughs (*p* = 0.03) and black and Asian groups than White ethnicities (*p* = 0.035), with a gradient by age (*p* = 0.005).

In relation to differences in the preferences for individual initiatives, Table 5 shows that patients from regular contract practices were significantly more likely to report that they would be influenced by vouchers for free treatment than those in the extended contract sites. In terms of dental attendance type, urgent attendees reported being significantly more influenced by reduced cost of treatment and dental leaflets/flyers. When those who reported paying for NHS services were compared with those who did not, the results suggested that free toothpaste, dental leaflets, an emergency dental helpline and flyers appear to be more likely to influence those who do not pay for NHS charges than those who had to pay. Banners outside practices were perceived as the most important influence on respondents who had moved home once, and dental posters on those who had moved home more than once, compared with those who had moved once only. Those who had moved more than once were also more influenced by free treatment vouchers than those who had never moved. All the above were significant at the 0.05 level. Further analysis shows differences in patient preferences between the two contract types and within patient groups as detailed in Table 5.

**Table 5:** Importance of influences by initiative and patient type

Patient group *n* = 450		Dental advertisements on buses	Dental posters at bus stops	Banners outside dental practices	Free toothpaste packets fornew patients	Vouchers for reduced cost of treatment	Vouchers for free treatment	Dental advertisements in newspapers	Dental leaflets/flyers	Emergency out of hours helpline	Dental article in a magazine	Removable helpline card in a magazine	PCT patient advisory liaison services
Sex *n* = 415	Male	3.3 (1.2)	3.3 (1.2)	3 (1.3)	3.1 (1.3)	2.5 (1.4)	2.4 (1.4)	3.2 (1.2)	3.2 (1.2)	2.8 (1.2)	3.3 (1.3)	3.3 (1.2)	3.3 (1.2)
	Female	3.1 (1.2)	3.1 (1.2)	2.8 (1.2)	2.7 (1.3)*	2.2 (1.3)*	2.1 (1.2)*	2.9 (1.2)*	2.9 (1.2)*	2.5 (1.1)*	2.9 (1.2)*	3 (1.2)*	2.9 (1.2)*
Age *n* = 415	16–24	**3 (1.1)**	**3 (1.2)**	2.8 (1.1)	**2.5 (1.3)**	**2.2 (1.2)**	2 (1.1)	2.9 (1.1)	**2.7 (1.0)**	2.8 (0.9)	3 (1.1)	**3.2 (1.2)**	3.2 (1.2)
	25–34	3.2 (1.2)	3.2 (1.1)	2.8 (1.2)	**2.7 (1.3)**	**2.2 (1.3)**	2 (1.2)	3 (1.1)	2.9 (1.1)	2.6 (1.2)	3.1 (1.1)	**3 (1.2)**	3.1 (1.2)
	35–44	**3 (1.1)**	**3 (1.1)**	2.7 (1.1)	**2.7 (1.2)**	**2.2 (1.2)**	**2.1 (1.2)**	**2.7 (1.1)**	**2.7 (1.1)**	2.6 (1.2)	2.8 (1.2)	**2.8 (1.1)**	2.8 (1.1)
	45–54	3.3 (1.3)	3.2 (1.2)	2.8 (1.3)	2.9 (1.4)	**2.2 (1.2)**	2.2 (1.3)	3.2 (1.3)	3 (1.2)	2.6 (1.3)	3 (1.4)	**3.1 (1.3)**	3.1 (1.2)
	55–64	3.4 (1.2)	3.4 (1.3)	3.3 (1.4)	**3.4 (1.3)**	**3 (1.4)**	**2.8 (1.5)**	**3.4 (1.4)**	**3.5 (1.3)**	2.9 (1.5)	3.3 (1.3)	**3.3 (1.4)**	3.2 (1.4)
	65–74	3.5 (1.1)	3.5 (1.2)	3.2 (1.4)	3.3 (1.3)	2.5 (1.6)	2.4 (1.6)	**3.5 (1.1)**	3.3 (1.2)	2.6 (1.3)	3.1 (1.4)	**3.4 (1.2)**	3.2 (1.3)
	75 and over	**4.4 (1.2)**	**4.4 (1.1)**	3.6 (1.7)	4 (1.2)	2.9 (1.7)	3.1 (1.6)	**4.1 (1.4)**	4.1 (1.1)	2.6 (1.6)	4 (1.2)	**4.1 (1.2)**	3.4 (1.3)
Type of current care *n* = 445	Urgent	3 (1.2)	3.2 (1.1)	3(1.2)	2.8 (1.3)	2.1 (1.3)*	1.9 (1.3)*	3 (1.1)	2.8 (1.1)*	2.7 (1.2)	3.1 (1.1)	3.1 (1.2)	3.1 (1.1)
	Regular attenders	3.4 (1.2)	3.1 (1.2)	2.9 (1.2)	2.8 (1.4)	2.7 (1.4)	2.7 (1.4)	3.3 (1.3)	3.(1.1)	2.8 (1.3)	3. (1.2)	3.4 (1.3)	3.3 (1.3)
Contract type *n* = 450	Regular	3 (1.2)	3.2 (1.2)	2.8 (1.3)	2.8 (1.3)	2.2 (1.2)*	2.1 (1.3)	3.1 (1.2)	3 (1.2)	2.5 (1.3)	3 (1.2)	3.1 (1.3)	3.1 (1.1)
	Extended	3.2 (1.2)	3.4 (1.3)	3.1 (1.4)	3. (1.2)	2.5 (1.3)	2.4 (1.4)	3.2 (1.2)	3.2 (1.2)	2.8 (1.4)	3.4(1.3)	3. (1.2)	3.1 (1.2)
Pay for NHS treatment *n* = 369	Pays	3.3 (1.2)	3.3 (1.2)	3 (1.2)	3 (1.3)	2.2 (1.3)	2.1 (1.3)	3 (1.2)	3 (1.1)	2.8 (1.3)	3.1 (1.2)	3.2 (1.2)	3.1 (1.2)
	Does not pay	3.1 (1.2)	3 (1.2)	2.8 (1.2)	2.6 (1.3)*	2.1 (1.3)	2.4 (1.4)	3.0 (1.2)	2.8 (1.2)*	2.4 (1.2)*	2.9 (1.2)	3 (1.3)	3.0 (1.3)
Number of times moved houses *n* = 362	More than once	3.6 (1.1)	3.6 (1.1)	3.1 (1.4)	**2.8 (1.4)**	2.5 (1.4)	**2.4 (1.5)**	3.3 (1.2)	3.1 (1.2)	2.9 (1.5)	3.2 (1.2)	3.3 (1.3)	3.4 (1.3)
	Once	3.0 (1.1)	3 (1.1)	2.6 (1.1)	**2.6 (1.2)**	2.1 (1.2)	**1.8 (1.0)**	2.8 (1.1)	2.7 (1.1)	2.4 (1.1)	2.7 (1.1)	3 (1.2)	3.0 (1.2)
	Never	3.3 (1.2)	3 .2 (1.2)	3.0 (1.2)	**3.0 (1.3)**	2.4 (1.3)	**2.2 (1.3)**	3.2 (1.2)	3.1 (1.2)	2.6 (1.2)	3.1 (1.2)	3 (1.3)	3.1 (1.2)
All patients		3.3 (1.2)	3.3 (1.2)	3 (1.2)	2.9 (1.3)	2.4 (1.3)	2.3 (1.3)	3.1 (1.2)	3.1 (1.2)	2.7 (1.2)	3.1 (1.2)	3.1 (1.2)	3.(1.2)

(1) The lower the score the more the group is influenced by an initiative. (2) Mean (SD). (3) Significant differences within patient *age groupings* and *number of times moved houses* are in bold; between gender and attendance groupings * identifies the group more influenced at *p* < 0.05

The analysis of age group versus awareness and influence of initiatives required a post-hoc analysis and suggested that dental advertisements on buses and dental posters at bus stops influenced 35–44 year olds significantly more than 16–24 year olds and those over 75 years. Overall, there was a trend towards younger adults considering that free toothpaste packs, reduced or free cost of treatment and dental leaflets/flyers being considered as significantly more influential (*p* = 0.05). Emergency out-of-hours helplines were considered influential, and equally so across age-groups, and this is important amongst older adults who reported less influence of initiatives on their dental attendance.

#### Barriers to dental treatment

**Fig. 1:**
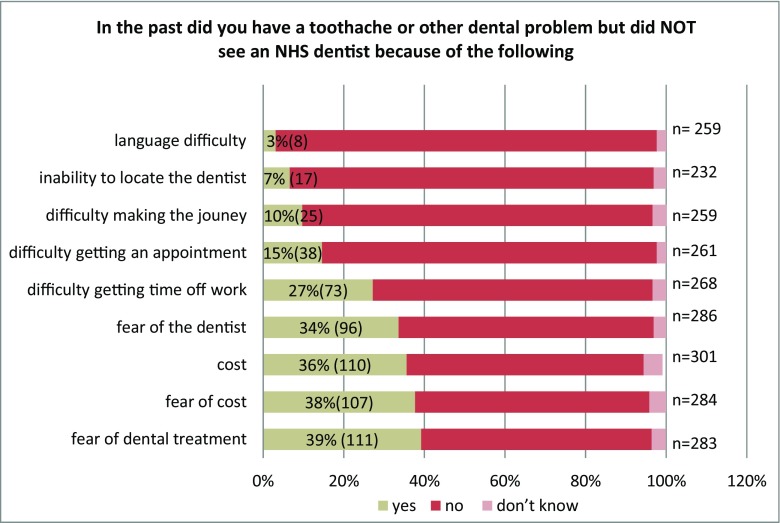
Reported barriers to dental care for attendees in three inner metropolitan boroughs

Although these adults were attendees, barriers to dental care were explored (Fig. 1). The most common barrier was fear of treatment (39%, *n* = 111), followed by cost (38%, 110) and fear of cost (38%, *n* = 107). Additional barriers involved difficulty in getting time off work (36%, *n* = 73), getting an appointment (34%, *n* = 38) and locating an NHS dentist in the past (7%, *n* = 17).

## Discussion

This research provides insight into the multiple initiatives to improve access to dental care across three inner city boroughs in London and their influence on patients. Vouchers for free treatment were perceived as the most influential option according to patients attending during this period; this is in line with the suggestions made by Boyce et al. (2008), who suggest financial incentives are the most effective in influencing individual choices and are likely to be most effective when used as one element of a wider programme to promote long-term behaviour change. Our findings suggest that location was reported as the most influential in patient’s choice of practice, particularly amongst men; women on the other hand were more likely to attend a practice based on its reputation. Gender-based differences are increasingly recognised as important considerations in developing and providing health services (Department of Health 2008).

### Influence of initiatives

Overall, some of the initiatives were perceived as much more influential than the others with differences in perception shown by age, sex and attendance behaviour. Vouchers for free treatment were perceived as the most influential with an average score of 3.07 (SD 1.17); however, in relation to awareness they were only ranked the 5th highest out of 12. Conversely, although individuals appear aware of leaflets, they were not reported to be influential on patients’ attendance pattern. It appeared that patients were less aware of initiatives they perceived would influence them to attend dental services. This can be explained by the fact that these initiatives, which addressed cost and urgent care, although provided locally, were not available throughout the access improvement programme. Interestingly, mobile information sources from other areas of London (notably buses with dental advertising), which passed regularly through the borough, as previously reported by Anderson and Morgan (1992), were amongst the lowest perceived influence; thus the investment in such advertising should be avoided in future. It is essential to note that the items individuals reported as influential correspond with addressing reported barriers (Borreani et al. 2008; Heaton et al. 2004; Hill et al. 2013). These commonly reported barriers were also similar to the 2009 London survey (Pau and Gallagher 2009), which found the reasons for not attending the dental practice in the last 12 months were linked to personal circumstances rather than difficulties in obtaining access or a lack of information.

In considering the survey findings, the representativeness of respondents must be considered carefully. The majority of respondents had been attending the practice for over 2 years (64%, *n* = 284), with only 7% (*n* = 29) reporting attending for the first time. This is consistent with national findings, whereby 61% of those attending were established patients for over 5 years and 14% were first time attendees (Hill et al. 2013). However, one in five patients reported attending as a dental emergency, as opposed to routine or planned work. This is higher than the Adult Health Survey 2009 where 64% reported being regular attenders (Hill et al. 2013), compared with 79% in this survey, and therefore consistent with being a socially deprived area.

Given local uptake of dental care, which more or less parallels the national level amongst adults, the above arguments seem reasonable. However, given the high levels of population turnover in the boroughs, the long-term relationship of these adults with the practices could be considered high. This suggests that the survey respondents were probably mainly from their regular patient base. Issues with accommodation and affordability of care were reported in this study, in line with the work of Steele (2009), in his independent review of NHS dental services in England and past local surveys (Al-Haboubi et al. 2013); they involved requests for longer opening hours and patient charges to be displayed.

The findings suggest that initiatives to promote dental attendance appealed to patient groups differently; although, given the response rate, differences between the types of practice must be treated with caution. There were, however, significant differences in the awareness of initiatives by different patient groups, with those attending for routine/planned visits being more aware of initiatives and similarly those with a past dental attendance behaviour, which involved regular visits being more aware of initiatives. Furthermore, there were significant differences in relation to the overall influence of initiatives by demography with Asians and Other ethnic groups significantly more influenced by initiatives in general than people of White ethnicity, and females being more influenced than males. The latter is in line with wider evidence, as females have been shown to have better health-seeking behaviour than males in nationwide surveys (NHS Digital 2011a, c), and locally in South East London (Al-Haboubi et al. 2013).

Limitations of the study include the response rate being slightly lower than ideal to compare types of practice and the fact that those who were questioned had already attended the dentist. Nonetheless, it was decided to survey practice attendees in this study as it validated their actual dental attendance. It is important to note that significant resources were poured into this campaign, in terms of both commissioning additional practice capacity and promoting services. Although dental access rates did increase in the area in the period after the initiatives (NHS Digital 2012), this can only be ecologically attributed to the initiatives as other wider societal factors occurring at the time may have played a role and initiatives in other parts of the country as this was a national move to address public concerns and actively promote NHS dentistry.

Emphasis by government on providing information for patients and the public to make choices about their care (Department of Health 2010), is commendable and future initiatives located in inner city areas should ensure that the key barriers are addressed, particularly financial incentives for those who are required to make co-payments (Minister for Health 2016). Initiatives specifically targeted to the relevant populations are likely to be successful in raising dental awareness should they be implemented; however, it may be of value to conduct focussed studies prior to instituting initiatives prior to roll out. By promoting dental attendance, as with this and past initiatives, we may improve awareness and uptake of care for those who might otherwise be ‘too busy to attend’ or those who perceive themselves to have ‘no need’, as demonstrated in the West Midlands by Anderson and Morgan (1992), over 2 decades ago. However, we may not affect those who are harder to reach. The findings of this study should prove helpful toplanners and commissioners of dental care on future social marketing strategies. Additionally, the research instrument may be used at varying points in planning, and monitoring initiatives, drawing on the opinions of the general public as well as those who successfully attend for dental care.

## Conclusion

Adult patients attending primary dental care in three inner-city metropolitan boroughs, during a period when dental care was actively promoted and uptake increased, appear generally to have a long-term relationship with their dental practice. Certain initiatives are perceived to facilitate access more effectively in sub-sections of the populations with financial incentives appearing to have the most attractive influence amongst this socially deprived community, and dental advertisements least. On the other hand, many adult patients certainly appear to be aware of banners located outside dental practices and of dental advertisements in magazines; however, their role in influencing attendance is debatable. Overall it is apparent that dental practices should clearly display their status, charges and emergency helpline numbers for NHS dental care in support of patients.
